# An Evidence-Based Review of Alternating Electric Fields Therapy for Malignant Gliomas

**DOI:** 10.1007/s11864-015-0353-5

**Published:** 2015-07-05

**Authors:** Eric T Wong, Edwin Lok, Kenneth D. Swanson

**Affiliations:** Brain Tumor Center and Neuro-Oncology Unit, Beth Israel Deaconess Medical Center, Harvard Medical School, 330 Brookline Avenue, Boston, MA 02215 USA; Department of Physics, University of Massachusetts in Lowell, Lowell, MA USA

**Keywords:** Alternating electric fields, Malignant gliomas, Glioblastoma, Review

## Abstract

Glioblastoma is a deadly disease and even aggressive neurosurgical resection followed by radiation and chemotherapy only extends patient survival to a median of 1.5 years. The challenge in treating this type of tumor stems from the rapid proliferation of the malignant glioma cells, the diffuse infiltrative nature of the disease, multiple activated signal transduction pathways within the tumor, development of resistant clones during treatment, the blood brain barrier that limits the delivery of drugs into the central nervous system, and the sensitivity of the brain to treatment effect. Therefore, new therapies that possess a unique mechanism of action are needed to treat this tumor. Recently, alternating electric fields, also known as tumor treating fields (TTFields), have been developed for the treatment of glioblastoma. TTFields use electromagnetic energy at an intermediate frequency of 200 kHz as a locoregional intervention and act to disrupt tumor cells as they undergo mitosis. In a phase III clinical trial for recurrent glioblastoma, TTFields were shown to have equivalent efficacy when compared to conventional chemotherapies, while lacking the typical side effects associated with chemotherapies. Furthermore, an interim analysis of a recent clinical trial in the upfront setting demonstrated superiority to standard of care cytotoxic chemotherapy, most likely because the subjects’ tumors were at an earlier stage of clonal evolution, possessed less tumor-induced immunosuppression, or both. Therefore, it is likely that the efficacy of TTFields can be increased by combining it with other anti-cancer treatment modalities.

## Introduction

Tumor treating fields (TTFields) represent a novel treatment modality for cancer that utilizes alternating electric fields at an intermediate frequency of 200 kHz. At this specific frequency, TTFields have been shown to penetrate into the head from the surface of the scalp. Computational modeling also showed that the fields are distributed inhomogeneously within the supratentorial regions of the brain, and they tend to become intensified near the ventricles [1•]. At the cellular level, the electromagnetic energy perturbs proteins that have large dipole moments. Cells treated with TTFields exhibited a variety of abnormalities indicative of mitotic catastrophe and aberrant mitotic exit, including cells in polyploidy prophase, rosettes, multi-spindled metaphase, single-spindled metaphase, and asymmetric anaphase [2]. Indeed, cells exhibit violent membrane blebbing as they enter anaphase and attempt to divide. This results in aberrant mitotic exit and subsequent cell death [3••]. Some of the proteins that are critical for the proper progression through mitosis have sufficiently high dipole moments to suggest that they may be targets of TTFields, including the mitotic septin complex and the α/β-tubulin monomeric subunit of tubulin. Septins constitute a family of GTP-binding proteins and septin 2, 6, and 7 oligomerize into a heterotrimer with an extremely large dipole moment of 2711 Debyes [4]. Importantly, this septin complex is required for functions that are necessary for the later stages of cell division. Septin 2, 6, and 7 heterotrimers rapidly polymerize and structurally organize within the cytokinetic furrow as cells exit metaphase. Once it is recruited, it then organizes contractile elements within the cytokinetic furrow above the equatorial cleavage plane by binding to F-actin filaments and spatially regulates myosin activation. RNAi-directed depletion of septin subunits of the heterotrimer results in mitotic catastrophe similar to that seen when cells attempt to divide in the presence of TTFields [5]. We have shown that TTFields disrupt the ability of septins to re-localize to the cytokinetic furrow and reduce the accumulation of F-actin [3••]. Therefore, TTFields affect tumor cells by interfering with their ability to complete mitosis by exerting electromagnetic induction forces that interfere with the function of proteins with high dipole moments [2, 3••].

TTFields therapy has been shown to have equivalent efficacy when compared to the best physician’s choice chemotherapy in a registration phase III clinical trial for recurrent glioblastoma [6]. This led to the FDA approval on April 8, 2011 for recurrent glioblastoma [Http://Www.Accessdata.Fda.Gov/Cdrh_Docs/Pdf10/P100034a.Pdf]. Interim analysis of the most recent phase III study in the newly diagnosed setting showed a significant improvement of outcomes leading to a crossover of subjects from the control arm to the experimental arm of the trial [7]. Here, we review our current understanding of the mechanisms of TTFields therapy, particularly from the physics and cell biology perspectives, as well as the available clinical data when it is applied to the treatment of glioblastoma.

## Electric field distribution within the brain

At a frequency of 200 kHz, the electric fields from the surface of the scalp can permeate into the brain. This is because the penetration of electromagnetic waves through any medium is frequency dependent. Past analyses have shown that the permittivity values were similar among the calvarial bone, gray matter, and white matter, while the conductivity values varied somewhat among these three structures [8].

The electric field intensity was directly measured in a patient receiving TTFields therapy while undergoing surgery for obstructive hydrocephalus from a large pineal meningioma at the Rambam Medical Center in Haifa, Israel. The measured intensity of electric field was validated to within 10 % of the simulated value using finite element method simulation [9].

**Fig. 1:**
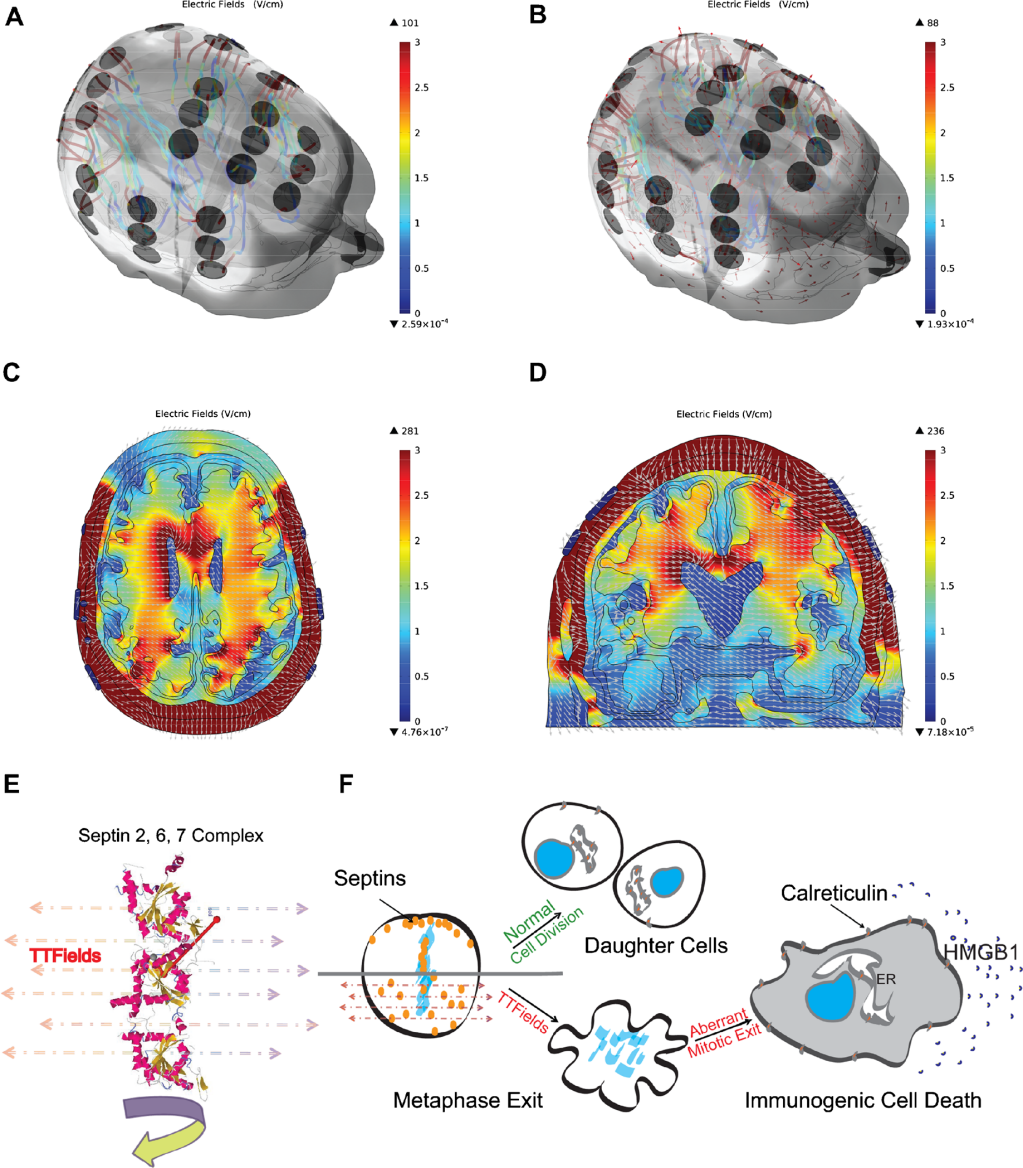
A 3-dimensional render of a human head with TTFields clinically applied via electrode arrays on a glioblastoma patient whose gross tumor volume is on the right side. **a** Streamlines showing the magnitude of the electric field and direction of the current emanating from each electrode on the surface of the scalp. **b** *Red arrows* indicating vector field of the electric field distribution inside the brain. The intracranial electric fields are displayed in **c** axial and **d** coronal planes. **e** TTFields induce a force on the septin 2, 6, and 7 complex that has an extremely large dipole moment of 2711 Debyes. **f** This results in mitotic catastrophe and aberrant mitotic exit, leading to an increased cell surface expression of the endoplasmic reticulum chaperonin calreticulin and the secretion of HMGB1 that acts as a danger signal when release from cells, both of which are essential for immunogenic cell death.

Using finite element analysis, 3-dimensional mapping of the electric field distribution within the brain revealed inhomogeneous distribution of the fields, with a higher field strength near the ventricular horns that is most likely a result of the high conductivity of the cerebrospinal fluid (Fig. 1).

## Cell biology effects of alternating electric fields on dividing tumor cells

TTFields disrupt the mitotic process in dividing tumor cells that results in violent membrane blebbing [3••, 10]. This results in the disordering of chromosomes from the metaphase plate during late metaphase or early anaphase, followed by aberrant mitotic exit in the absence of cytokinesis resulting in multinucleated cells and subsequent apoptosis [3••].

The septin 2, 6, and 7 family members heterotrimerize into a protein complex that possesses an extremely large dipole moment of 2711 Debyes, and it is active in mitosis [4]. This complex serves to regulate contractile function within the cytokinetic furrow, and it is likely to provide tensile strength needed within the submembranous cortical cytoskeleton to restrain the hydrostatic pressures within the cytoplasm during cell division. It has been shown to be a target of alternating electric fields, and the disruption of this protein results in disordered segregation of chromosome and cytoplasmic contents [3••].

Following TTFields-induced aberrant mitotic exit, cells exhibit signs of cellular stress that mark them for immune destruction and facilitate immune activation. Specifically, this type of cellular stress causes increased cell surface expression of the endoplasmic reticulum chaparonin calreticulin and the secretion of HMGB1 that acts as a danger signal when released from cells [11]. The presence of calreticulin on the plasma membrane is also seen in virally infected cells, as well as tumor cells exposed to certain chemotherapy agents [12]. This has been termed “immunogenic cell death” to differentiate it from apoptosis, which is immunosuppressive. Immunogenic cell death leads to tumor destruction.

There is a compelling evidence that TTFields increase the anti-tumor immunogenicity in vivo. When highly metastatic VX-2 tumors were injected into the kidney capsule of rabbits and treated with TTFields for 7 days then allowed to grow for an additional 21 days, the number of pulmonary metastases was significantly reduced when compared to untreated control animals [13]. When the lung metastases were recovered from animals, there was increased infiltration of immune cells in the TTFields-treated metastases as compared with the non-treated ones [14].

## Treatment

The management of malignant gliomas should be undertaken in a multimodal fashion, with neurosurgical input, radiation oncology expertise, and chemotherapy administration. Now, with the availability of alternating electric fields therapy as a fourth modality of treatment, neuro-oncologists will need to factor in this therapy within the spectrum of available treatments. For newly diagnosed malignant gliomas, maximal safe neurosurgical resection is still recommended and resection accomplishes two goals of establishing a histological diagnosis and achieving cytoreduction. Although it has not been subjected to a randomized clinical trial, the best evidence for a benefit of cytoreduction is based on a retrospective analysis showing a 4.2-month survival advantage in patients with at least a 98 % resection versus those with less than 98 % [15]. However, if safe resection is not possible, biopsy to obtain a histological diagnosis is still indicated. Once a diagnosis of glioblastoma is established, patients proceed to standard of care treatment, which consists of external beam, involved-field cranial radiotherapy plus concomitant daily temozolomide, followed by 6 cycles of adjuvant temozolomide [16]. Alternatively, patients may be enrolled in a clinical trial at initial diagnosis and, depending on the conduct of the trial, may either receive treatment independently or in conjunction with standard of care treatment. Although upfront treatment can provide a period of stabilization for the glioblastoma, recurrence is the rule and additional treatments are typically needed to control tumor progression, alleviate neurological deficits, or both.

At the time of tumor recurrence, patients with a Karnofsky performance score of 70 or higher may be eligible for clinical trials. Those who are ineligible can be treated with single-agent bevacizumab or TTFields therapy since both were approved by the FDA for recurrent glioblastoma in 2009 and 2011, respectively. The benefit of bevacizumab was based on two single-arm phase II studies demonstrating a radiographic response rate of 30–40 % [17, 18]. However, infiltrative glioblastoma is the typical pattern of relapse and salvage chemotherapy after bevacizumab failure only offered a median overall survival of 5.2 months and progression-free survival of 2.0 months [19]. Therefore, alternative treatments are desperately needed for this population and TTFields therapy was demonstrated to have equivalent efficacy when compared to chemotherapy in this setting [6]. However, the optimal use of this device and its combination with conventional treatments are awaiting further investigation. Here, we review the currently available clinical data when it is applied to the treatment of glioblastoma, which is also summarized in Table 1.

**Table 1:** Summary of clinical data on TTFields treatment for malignant gliomas

Phase III trial for newly diagnosed glioblastoma interim analysis	TTFields treatment + temozolomide	Temozolomide alone	Hazard ratio	*P*
Overall survival, median^a^	19.6 months	16.6 months	0.75	0.03
Progression-free survival^a^	7.1 months	4.0 months	0.63	<0.01
Phase III recurrent glioblastoma	TTFields treatment (*n* = 120)	Active chemotherapy (*n* = 117)		
Overall survival, median^b^	6.6 months	6.0 months	0.86 (95 % CI 0.66–1.12)	0.27
1-year survival	20 %	20 %		
2-year survival	8 %	4		
3-year survival	5 %	1 %		
Prognostic factors, median overall survival^c^				
Prior bevacizumab failure	6.0 months (*n* = 23)	3.3 months (*n* = 21)	0.43 (95 % CI 0.22–0.85)	0.02
Prior low-grade glioma	25.3 months (*n* = 12)	7.7 months (*n* = 9)	0.31 (95 % CI 0.09–0.99)	0.05
Tumor size ≥18 cm^2^	5.6 months (*n* = 39)	3.3 months (*n* = 41)	0.53 (95 % CI 0.32–0.85)	<0.01
Karnofsky performance status ≥80	7.9 months (*n* = 83)	6.1 months (*n* = 77)	0.71 (95 % CI 0.51–0.99)	0.05
TTFields treatment versus bevacizumab	6.6 months (*n* = 120)	4.9 months (*n* = 36)	0.64 (95 % CI 0.41–0.99)	0.05
Progression-free survival, median^b^	2.2 months	2.1 months	0.81 (95 % CI 0.60–1.09)	0.13
PFS at 6 months	21 %	15 %		
Responders^d^	14	7		
Response status, median overall survival				
Partial and complete response versus	24.7 months (*n* = 14)			
Stable disease	7.6 months (*n* = 34)		0.28 (95 % CI 0.14–0.58)	<0.01
Progressive disease	5.5 months (*n* = 59)		0.24 (95 % CI 0.14–0.42)	<0.01
Prognostic factor in TTFields treatment responders^e^				
Overall survival, median				
With prior low-grade glioma	27.7 months			
Without prior low-grade glioma	16.6 months			0.05
Daily dexamethasone dose, median				
Responders	1.0 mg			
Nonresponders	5.2 mg			<0.01
Cumulative dexamethasone dose, median				
Responders	7.1 mg			
Nonresponders	261.7 mg			<0.01
Treatment-related adverse events, ≥grade 2^b,f^				
Hematological	3 %	17 %		
Gastrointestinal	4 %	17 %		
Dermatological	2 %	0 %		
Nervous system disorders	30 %	28 %		
Recurrent glioblastoma from patient registry data set (PRiDe)	PRiDe TTFields treatment (*n* = 457)	EF-11 TTFields treatment (*n* = 120)		
Survival^g^				
1-year survival	44 %	20 %		
2-year survival	30 %	9 %		
Prognostic factors, median overall survival^g^				
Number of prior recurrences				
First recurrence versus	20 months			
Second recurrence	8.5 months, HR = 0.6 (95 % CI 0.4–0.9), *P* = 0.03			
Third to fifth recurrence	4.9 months, HR = 0.3 (95 % CI 0.2–0.5), *P* < 0.01			
Compliance				
<75 % versus	4.0 months			
≥75 %	13.5 months, HR = 0.4 (95 % CI 0.3–0.6), *P* < 0.01			
Karnofsky performance status				
90–100 versus	14.8 months			
70–90	7.7 months, HR = 0.6 (95 % CI 0.4–0.9), *P* < 0.01			
10–60	6.1 months, HR = 0.4 (95 % CI 0.2–0.6), *P* < 0.01			
Prior bevacizumab use				
No versus	13.4 months			
Yes	7.2 months, HR = 0.5 (95 % CI 0.4–0.7), *P* < 0.01			
Prior debulking surgery				
No versus	8.9 months			
Yes	9.8 months, HR = 1.1 (95 % CI 0.8–1.5), *P* = 0.79			

^a^Stupp R, Wong E, Scott C, et al. Neuro-Oncol 2014;16(Suppl 5):v167

^b^Stupp R, Wong ET, Kanner AA, et al. Eur J Cancer 2012;48:2192-2202

^c^Kanner AA, Wong ET, Villano JL, et al. Semin Oncol 2014;41(Suppl 6):S25-S34

^d^Vymazal J, Wong ET. Semin Oncol 2014;41(Suppl 6):S14-S24

^e^Wong ET, Lok E, Swanson KD, et al. Cancer Med 2014;3:592-602

^f^Lacouture ME, Davis ME, Elzinga G, et al. Semin Oncol 2014;41(Supple 4):S1-S14

^g^Mrugala MM, Engelhard HH, Tran DD, et al. Semin Oncol 2014;41(Supple 6):S4-S13

## TTFields therapy for recurrent glioblastoma

At present, the only indication approved by the FDA is for the treatment of recurrent glioblastoma. This is based on the registration phase III clinical trial (ClinicalTrials.gov: NCT00379470) demonstrating equivalent efficacy between TTFields therapy and best physician’s choice chemotherapy (based on the best available treatment as offered by the treating physician) [6].

The primary endpoint of the trial was overall survival, and the median overall survival was 6.6 months for TTFields (*n* = 120) versus 6.0 months for the best physician’s choice chemotherapy (*n* = 117), with a hazard ratio of 0.86 (95 % CI 0.66–1.12; *P* = 0.27). It is notable that 31 % of the BPC cohort received bevacizumab alone or in combination with chemotherapy. The median progression-free survival of TTFields and the best physician’s choice chemotherapy was 2.2 and 2.1 months, respectively, with a hazard ratio of 0.81 (95 % CI 0.60–1.09; *P* = 0.16), and the progression-free survival at 6 months was 21.4 % (95 % CI 13.5–29.3) and 15.1 % (95 % CI 7.8–22.3), respectively (*P* = 0.13). One year survival rate was 20 % in both cohorts.

The most common toxicity associated with the device was grade 1 or 2 scalp irritation at a rate of 16 %, but none had severity of grade 3 or 4. The scalp irritation can be managed by applying topical corticosteroid and by shifting of the arrays slightly during each array exchange [20]. The most important advantage associated with the TTFields therapy device, when compared to chemotherapy, is that it has far fewer grade 2 or greater hematological toxicities, 3 versus 17 %, respectively, and fewer adverse gastrointestinal events, 4 versus 17 %, respectively.

Analysis of quality of life demonstrated that patient treated with the device had better cognitive and emotional functions than those treated with chemotherapy while appetite loss, constipation, diarrhea, fatigue, nausea, vomiting, and pain were more often seen in patients treated with chemotherapy. Based on the equivalent efficacy results and the lack of serious toxicities, the TTFields therapy device was approved by US FDA on April 8, 2011 for the treatment of recurrent glioblastoma.

*Post hoc* analysis showed that a higher proportion of responders had secondary glioblastoma than nonresponders [21••]. Five of the 14 responders (36 %) treated with TTFields monotherapy had prior low-grade histology while none of the seven responders (0 %) treated with the best physician’s choice chemotherapy did.

The analysis also showed that responders used less dexamethasone than nonresponders [21••]. In the TTFields therapy cohort, the median daily dexamethasone dose used was 1.0 mg for responders versus 5.2 mg for nonresponders (P=0.0019) and the median cumulative dexamethasone dose was 7.1 mg for responders versus 261.7 mg for nonresponders (P<0.0001). In the best physician’s choice chemotherapy cohort, the median daily dexamethasone dose used was 1.2 mg for responders versus 6.0 mg for nonresponders (P=0.0041) and the median cumulative dexamethasone dose was 348.5 mg for responders versus 242.3 mg for nonresponders (P=0.9520). These data suggest that concurrent dexamethasone use may influence the efficacy of TTFields therapy.

## TTFields therapy as used in clinical practice

Patients who received treatment from the TTFields device in clinical practice may have different clinical characteristics and outcomes from those who participated in the registration trial. To determine whether or not this is the case, a patient registry dataset (PRiDe) was developed in an effort to capture clinical practice data pertaining to the use of TTFields therapy. At the time of publication, this dataset included 457 patients from 91 treatment centers in the USA [22•].

The median OS was 9.6 months among patients treated in PRiDe as compared to 6.6 months in the TTFields monotherapy arm in the phase III trial while the 1-year OS rate was also longer at 44 % as compared to 20 %, respectively [6, 22•]. It is important to note that some patients in PRiDe may have used other treatments, such as conventional cytotoxic chemotherapy, bevacizumab, or even alternative medicine, in conjunction with TTFields therapy, but this aspect of treatment was not adequately captured because this dataset is from a registry.

About 33 % of patients at their first glioblastoma recurrence used TTFields therapy as compared to only 9 % in the registration phase III clinical trial [22•]. Favorable prognostic factors for patient survival include treatment with TTFields therapy at first or second relapses versus third or later recurrences, as well as no prior bevacizumab use [22•].

## TTFields therapy for newly diagnosed glioblastoma

TTFields therapy is currently being tested in a randomized phase III clinical trial for subjects with newly diagnosed glioblastoma (NCT0916409). The goal of this study is to compare the efficacy of TTFields plus adjuvant temozolomide versus adjuvant temozolomide alone by randomizing the subjects to the respective treatment arms in a 2:1 fashion, after the completion of initial treatment with radiation and concomitant daily temozolomide [16]. The primary endpoint is progression-free survival, and the secondary endpoints are overall survival, progression-free survival at 6 months, survival at 1 and 2 years, as well as quality of life assessment. So far, all 700 pre-specified subjects have been enrolled and randomized.

In a pre-specified interim analysis of the first 315 subjects after a minimum follow-up of 18 months, the intent-to-treat cohort received TTFields plus temozolomide (*n* = 210) had a longer progression-free survival than the cohort treated with temozolomide alone (*n* = 105), median 7.1 (95 % CI 5.9–8.2) months versus 4.0 (95 % CI 3.0–4.3) months (HR = 0.63, Log rank *P* = 0.0014). The median overall survival also favors the TTFields plus temozolomide group, 19.6 (95 % CI 16.5–24.1) months versus 16.6 (95 % CI 13.5–19.1) months, respectively (HR = 0.75, Log rank *P* = 0.034), as well as the per protocol population that started the second cycle of treatment, 20.5 (95 % CI 16.5–24.1) months (*n* = 196) versus 15.5 (95 % CI 13.5–19.1) months (*n* = 84), respectively (HR = 0.67, Log rank *P* = 0.0042).

There were no unexpected adverse events between the TTFields plus temozolomide and the temozolomide alone cohorts, and respective grade 3 and 4 hematological toxicities (12 versus 9 %), gastrointestinal disorders (5 versus 2 %), and convulsions (7 versus 7 %) were similar. Scalp reaction, however, was more common in the device-treated cohort, 49 % for grades 1 and 2 as well as 7 % for grade 3 and 4 toxicities, than the temozolomide-only cohort, 5 % for grade 1 and 2 toxicities as well as 5 % for grade 3 and 4 toxicities.

The follow-up of the remaining trial subjects will most likely mature in another year such that final data from the trial will be available by the end of 2016.

## Additional investigational studies of TTFields therapy for the central nervous system or other malignancies

Combinations with TTFields are being studied in patients with recurrent glioblastoma including bevacizumab (NCT01894061) and bevacizumab together with hypofractionated stereotactic irradiation (NCT01925573).

TTFields therapy is currently being investigated in patients with other types of central nervous system malignancies, including its use for recurrent atypical and anaplastic meningiomas (NCT01892397), as well as in those patients with 1–5 brain metastases from non-small cell lung cancer (NCT01755624).

TTFields therapy is also being investigated in systemic malignancies, including its use in combination with gemcitabine for advanced pancreatic adenocarcinoma (NCT01971281), in combination with paclitaxel in recurrent ovarian carcinoma (NCT02244502), as well as in combination with pemetrexed and cisplatin or carboplatin for malignant pleural mesothelioma (NCT02397928).

## References

[1.•] Miranda PC, Mekonnen A, Salvador R, Basser PJ (2014). Predicting the electric field distribution in the brain for the treatment of glioblastoma. Phys Med Biol.

[2] Kirson ED, Gurvich Z, Schneiderman R (2004). Disruption of cancer cell replication by alternating electric fields. Cancer Res.

[3.••] Gera N, Yang A, Holtzman T, Lee SX, Wong ET, Swanson KD. Tumor treating fields perturb the localization of septins and cause aberrant mitotic exit. PLOS One (2015) doi:10.1371/journal.pone.0125269.This study demonstrated that septin 2, 6 and 7 complexes likely constitute the major intracellular target of TTFields. It also demonstrated that mitotic disruption occurred during anaphase and resulted in aberrant mitosis and subsequent p53 dependent cell cycle arrest and apoptosis suggesting a possible role for individual patient tumor genetics in TTFields response.

[4] Felder CE, Prilusky J, Silman I, Sussman JL (2007). A server and database for dipole moments of proteins. Nucleic Acids Res.

[5] Gilden JK, Peck S, Chen YC, Krummel MF (2012). The septin cytoskeleton facilitates membrane retraction during motility and blebbing. J Cell Biol.

[6] Stupp R, Wong ET, Kanner AA (2012). NovoTTF-100A versus physician’s choice chemotherapy in recurrent glioblastoma: a randomised phase III trial of a novel treatment modality. Eur J Cancer.

[7] Stupp R, Wong ET, Scott C (2014). Interim analysis of the EF-14 trial: a prospective, multi-center trial of NovoTTF-100A together with temozolomide compared to temozolomide alone in patients with newly diagnosed GBM. Neuro-Oncology.

[8] Gabriel C, Gabriel S, Corthout E (1996). The dielectric properties of biological tissues: I. Literature survey. Phys Med Biol.

[9] Kirson ED, Dbaly V, Tovarys F (2007). Alternating electric fields arrest cell proliferation in animal tumor models and human brain tumors. Proc Natl Acad Sci U S A.

[10] Lee SX, Wong ET, Swanson KD (2011). Mitosis interference of cancer cells during anaphase by electric field from NovoTTF-100A. Neuro-Oncology.

[11] Chaput N, De Botton S, Obeid M (2007). Molecular determinants of immunogenic cell death: surface exposure of calreticulin makes the difference. J Mol Med (Berl).

[12] Obeid M, Tesniere A, Ghiringhelli F (2007). Calreticulin exposure dictates the immunogenicity of cancer cell death. Nat Med.

[13] Kirson ED, Schneiderman RS, Dbaly V (2009). Chemotherapeutic treatment efficacy and sensitivity are increased by adjuvant alternating electric fields (TTFields). BMC Med Phys.

[14] Kirson ED, Giladi M, Gurvich Z (2009). Alternating electric fields (TTFields) inhibit metastatic spread of solid tumors to the lungs. Clin Exp Metastasis.

[15] Lacroix M, Abi-Said D, Fourney DR (2001). A multivariate analysis of 416 patients with glioblastoma multiforme: prognosis, extent of resection, and survival. J Neurosurg.

[16] Stupp R, Hegi ME, Mason WP (2009). Effects of radiotherapy with concomitant and adjuvant temozolomide versus radiotherapy alone on survival in glioblastoma in a randomised phase III study: 5-year analysis of the EORTC-NCIC trial. Lancet Oncol.

[17] Friedman HS, Prados MD, Wen PY (2009). Bevacizumab alone and in combination with irinotecan in recurrent glioblastoma. J Clin Oncol Off J Am Soc Clin Oncol.

[18] Kreisl TN, Kim L, Moore K (2009). Phase II trial of single-agent bevacizumab followed by bevacizumab plus irinotecan at tumor progression in recurrent glioblastoma. J Clin Oncol Off J Am Soc Clin Oncol.

[19] Iwamoto FM, Abrey LE, Beal K, et al. Patterns of relapse and prognosis after bevacizumab failure in recurrent glioblastoma. Neurology 2009;73(15):1200–1206. 10.1212/WNL.0b013e3181bc0184 PMC2839807 19822869

[20] Lacouture ME, Davis ME, Elzinga G (2014). Characterization and management of dermatologic adverse events with the NovoTTF-100A System, a novel anti-mitotic electric field device for the treatment of recurrent glioblastoma. Semin Oncol.

[21.••] Wong ET, Lok E, Swanson KD, et al. Response assessment of NovoTTF-100A versus best physician’s choice chemotherapy in recurrent glioblastoma. Cancer Med. 2014;3(3):592–602. The post hoc analysis demonstrated the importance of the dose of concurrent dexamethasone used by subjects in the phase III trial that had an association with response to TTFields therapy. 10.1002/cam4.210 PMC4101750 24574359

[22.•] Mrugala MM, Engelhard HH, Dinh Tran D, et al. Clinical practice experience with NovoTTF-100A system for glioblastoma: the Patient Registry Dataset (PRiDe). Semin Oncol. 2014;41 Suppl 6:S4–13. This paper documented the pattern of TTFields therapy usage in clinical practice. 10.1053/j.seminoncol.2014.09.010 25213869

